# Selenium in Agricultural Products: Advances in Detection of Total Content and Speciation

**DOI:** 10.3390/foods15111927

**Published:** 2026-05-29

**Authors:** Yanan Yu, Liyuan Zhao, Chaohua Tang, Qingyu Zhao, Yuchang Qin, Junmin Zhang

**Affiliations:** State Key Laboratory of Animal Nutrition and Feeding, Institute of Animal Sciences, Chinese Academy of Agricultural Sciences, Beijing 100193, China

**Keywords:** total selenium, selenium speciation, agricultural products

## Abstract

Selenium (Se) is an essential trace element for humans, primarily obtained from dietary sources, particularly protein-rich foods. Owing to its narrow margin between nutritional requirement and toxicity, as well as the strong dependence of its bioavailability on chemical speciation rather than total concentration, accurate determination of Se in processed agricultural products is critically important. Organic Se species generally exhibit higher bioavailability, greater nutritional value, and lower toxicity compared to inorganic forms, highlighting the necessity of both total Se quantification and speciation analysis. This review critically evaluates recent advances in analytical methods for total Se determination and speciation, with emphasis on sample preparation, species stability, and factors affecting accuracy. While significant progress has been made in total Se analysis, speciation remains challenging due to low analyte levels, complex food matrices, species transformation during extraction, and the lack of standardized methods and certified reference materials. Consequently, combining total and speciation data is necessary for meaningful nutritional evaluation. Future work should focus on improving extraction protocols, enhancing the sensitivity of techniques such as HPLC-ICP-MS, and developing standardized methods for routine application in the food industry.

## 1. Introduction

The health benefits of selenium (Se) are numerous, and it is implicated in multiple aspects of health and disease [1,2]. Selenocysteine (Sec) is found in proteins and enzymes as the 21st naturally occurring amino acid. Twenty-five selenoproteins and enzymes containing Se have been discovered, including Selenoprotein P (SelP), T (SelT), M (SelM), glutathione peroxidase, thioredoxin, and iodothyronine deiodinase, contributing to antioxidant activity, anticancer activity, and immune system improvement [3,4]. Lack of Se in the human body is associated with premature aging, impaired sperm motility, Keshan disease, Kashin–Beck disease, and an increased risk of myocardial infarction [5,6,7]. However, it is crucial to emphasize that Se intake must be maintained within a reasonable range, as excessive consumption may lead to selenosis, causing adverse effects such as alopecia and dermatitis, and may even increase the risk of skin cancer and all-cause mortality [8,9,10]. Se is an essential trace element that cannot be synthesized endogenously by humans or other organisms and must be obtained from the diet. For humans, maintaining adequate Se levels through a balanced diet is the primary means of supporting health; however, its safety and efficacy are critically dependent on both its chemical species and intake dosage [6,11]. Se absorption in humans primarily occurs in the duodenum, with its bioavailability influenced by multiple factors. Organic Se exhibits higher bioavailability compared to inorganic Se [12]. As recommended by the US Food and Nutrition Board, the recommended dietary allowance for adults is 55 μg·day^−1^, while the tolerable upper intake level (UL) is 400 μg·day^−1^ in the USA [13]. According to the European Food Safety Authority (EFSA, 2023), the UL for Se in adults is 255 μg/day, indicating a relatively narrow margin between nutritional requirement and toxicity [14]. According to the Chinese Nutrition Society (2011), Se intake recommendations for healthy Chinese adults range from 60 to 400 μg·day^−1^ [15].

Dietary Se is obtained mainly from nuts (particularly Brazil nuts), seafood, animal products (e.g., beef, chicken, eggs, and cheese), cereals (corn, wheat, and rice), and legumes (soybeans), with these sources being listed approximately from higher to lower Se content. However, Se concentrations in plant-based foods may vary considerably according to geographic and soil conditions [16,17,18,19]. In those foods, Se appears in various forms, including selenite (Se(IV)) and selenate (Se(VI)) and several selenoamino acids, including selenomethionine (SeMet), selenocystine (SeCys_2_), selenohomocysteine (SeHoCys), se-methylselenocysteine (SeMeCys) and γ-glutamyl-Se-methylselenocysteine (γ-Glu-MeSeCys) [20]. Dietary Se intake varies significantly among populations worldwide due to marked differences in Se concentrations in foodstuffs. Se is unique in that it has a very narrow margin between toxicity and deficiency [13,21]. Therefore, monitoring Se concentrations in traditional and most commonly used foods is crucial in each country of the world.

**Figure 1:**
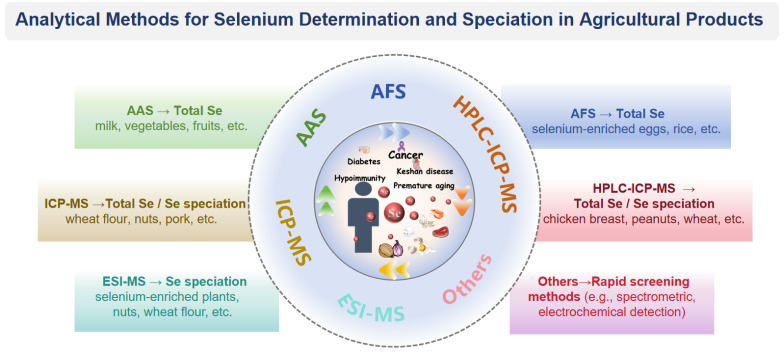
Main Analytical Techniques for Dietary Se. Arrows indicate method–measurement relationships, labels indicate sample types, and colors indicate analytical techniques. (Some of the material in the figure was taken from vecteezy.com.)

Determining the Se content of various foodstuffs is extremely important since there is a narrow safety gap between low and high levels. Recently, Pyrzynska [22] reviewed Se speciation analysis in foods, with particular attention to the bioavailability and safety of different Se forms. However, total Se determination and method comparison in processed agricultural products were not the main focus of that review. Therefore, the present review summarizes analytical methods for both total Se determination and Se speciation in agricultural products, with emphasis on sample preparation, species stability, matrix effects, and method applicability. This review summarizes the detection methods for Se in agricultural products, including total Se content and Se speciation detection (Figure 1), which schematically illustrates the classification and relationships of these techniques within the overall analytical framework, and each technique differs in sensitivity, selectivity, and applicability depending on analytical objectives. Currently available Se detection methods mainly include: (i) AFS (atomic fluorescence spectrometry); (ii) AAS (atomic absorption spectroscopy); (iii) ICP-MS (inductively coupled plasma mass spectrometry); (iv) HPLC-ICP-MS (high-performance liquid chromatography-inductively coupled plasma mass spectrometry); (v) ESI-MS (electrospray ionization mass spectrometry).

By acquiring in-depth insights into these analytical techniques, researchers can conduct more accurate assessments of Se levels in agricultural products, ensuring the safety of the food supply and maintaining optimal quality.

## 2. Literature Search Strategy

**Figure 2:**
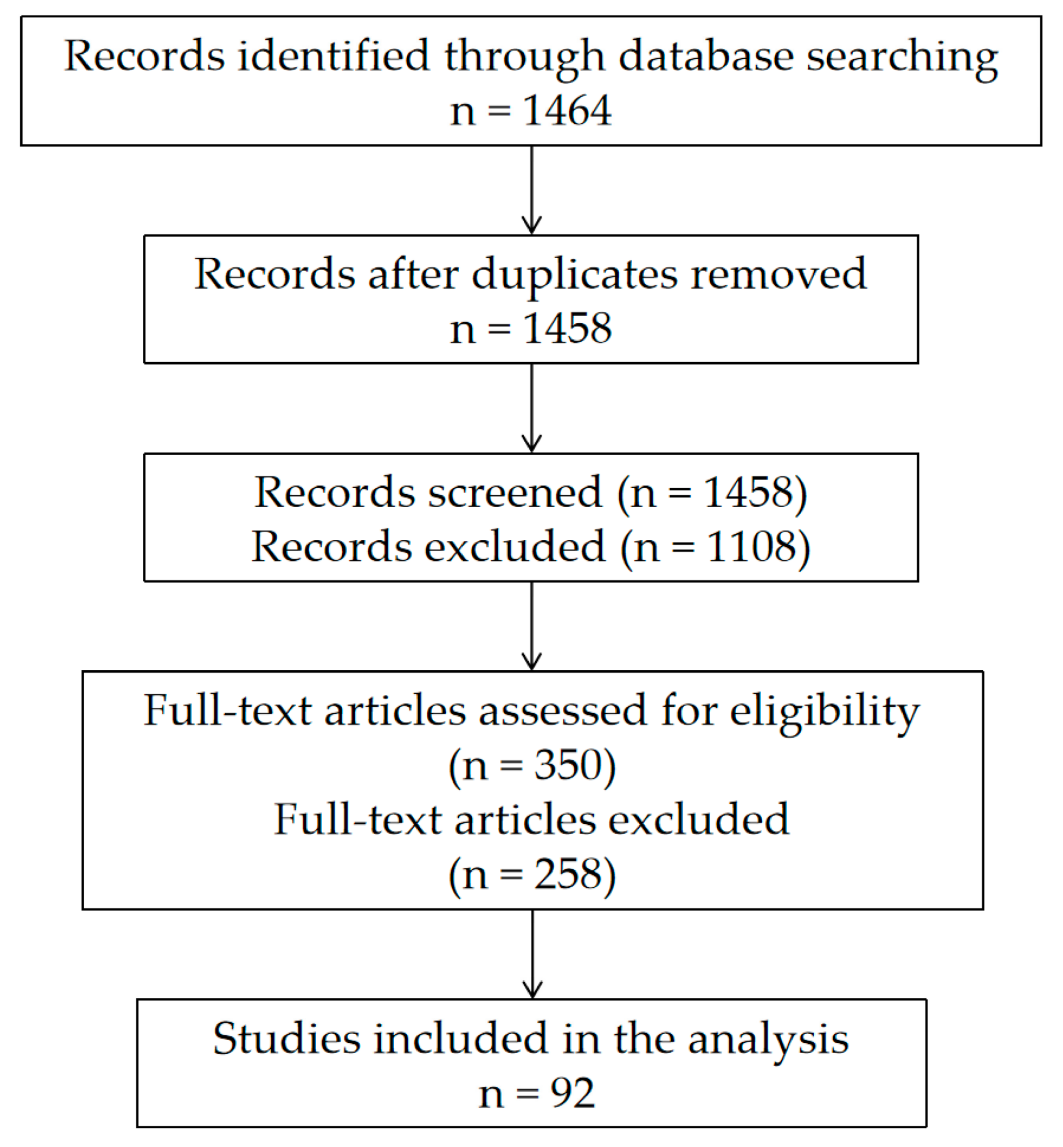
Flow diagram of the literature selection process.

A structured literature search was conducted in the Web of Science Core Collection on 24 April 2026, covering all publications from the database’s inception to that date. The search employed the following terms: (Selenium OR Se) AND (speciation OR “species analysis”) AND (food OR “agricultural product” OR “processed food”). Initially, 1464 records were retrieved. After removing duplicates, 1458 records remained for title and abstract screening. During this stage, 1108 records were excluded due to irrelevance to Se determination or speciation in food, processed food, or agricultural products. The full texts of the remaining 350 articles were then assessed according to predefined inclusion and exclusion criteria. Studies were included if they: (i) reported total Se determination or Se speciation analysis; (ii) focused on food or agricultural products, including processed food; and (iii) provided sufficient methodological details, such as sample preparation, analytical techniques, and validation parameters. After full-text assessment, 258 articles were excluded due to a lack of primary experimental data, irrelevant scope, or insufficient methodological information. Ultimately, 92 studies were included in the final analysis. The literature selection process, following the PRISMA guidelines, is illustrated in Figure 2, ensuring transparency and rigor in the selection procedure. Data extraction focused on analytical methods, sample preparation procedures, Se species identified, and factors influencing species stability and measurement accuracy.

## 3. Total Se Detection

Total Se serves as the most direct indicator of Se content in agricultural products. Among routine analytical methods, atomic fluorescence spectrometry (AFS) and atomic absorption spectrometry (AAS) remain widely adopted due to their operational simplicity and low cost [23]. AFS offers superior sensitivity, whereas AAS is more accessible in general laboratories. However, both techniques are confined to total Se measurement and are prone to matrix interference—limitations that constrain their applicability in complex sample matrices. Inductively coupled plasma mass spectrometry (ICP-MS) addresses some of these shortcomings by enabling highly sensitive, multi-element detection, making it the preferred choice for trace-level analysis. Its routine use, however, is constrained by higher operational costs and potential spectral interferences [24]. Other techniques, including spectrophotometry, fluorimetry, and electrochemical or nuclear methods, have largely fallen out of common use due to insufficient sensitivity or robustness. Sample preparation is equally critical for accurate quantification. For hydride generation-based approaches, complete mineralization—typically achieved via HNO_3_–H_2_O_2_ digestion—followed by the reduction of Se(VI) to Se(IV) is essential to enable hydride formation. Ultimately, while total Se analysis provides essential quantitative information, it does not capture Se speciation, which determines both bioavailability and toxicity [25,26,27].

The data compiled in Table 1 underscore the considerable variability in Se content across various agricultural products, a result of both natural background levels and intentional biofortification strategies. Analyses utilizing techniques such as HPLC-ICP-MS, HG-AFS, and d-CPE coupled with HG-AFS revealed this wide range: dairy products (e.g., milk) contained Se concentrations between 32.5 μg L^−1^ and 75.2 μg L^−1^, while fruits and vegetables like tomatoes and apples ranged from 26.7 μg kg^−1^ to 62.8 μg kg^−1^ [28,29]. Notably, certain products like cabbage and Se-enriched garlic exhibited exceptionally high concentrations, the latter reaching up to 952,000 μg kg^−1^ [30]. In cereals and legumes, Se content varied from 35.8 μg kg^−1^ to 288.1 μg kg^−1^ [31]. Eggs from poultry species were measured to contain between 374 μg kg^−1^ and 479 μg kg^−1^ of Se [32]. Seafood samples, including canned fish and shrimp, showed concentrations from 95.6 μg kg^−1^ to 175.3 μg kg^−1^ [31]. Other items, such as black pepper and spirulina, also demonstrated high levels, with black pepper reaching 11,100 μg kg^−1^ [33]. To ensure analytical accuracy, sample preparation predominantly relied on microwave digestion or cloud point extraction. These findings highlight the necessity for standardized methods to accurately quantify Se levels and evaluate the nutritional contribution of Se in foods.

### 3.1. Atomic Fluorescence Spectrometry

AFS is a highly sensitive and selective technique for the determination of trace elements. It is based on the absorption of element-specific radiation by atomic vapor, followed by fluorescence emission detected perpendicular to the excitation source [41]. When coupled with hydride generation (HG-AFS), the method provides ultra-low detection limits, a wide linear dynamic range, and strong resistance to matrix interference, while maintaining relatively simple operation and low cost [42].

HG-AFS has been widely applied to the determination of total Se in various agricultural and food samples, including eggs, rice, garlic, and tea, generally demonstrating satisfactory analytical performance in terms of sensitivity, precision, and recovery [43,44,45,46]. In addition, multi-element detection can be achieved in some configurations, further improving analytical efficiency.

In rice, Se predominantly exists in organic forms such as selenocysteine (Sec) and selenomethionine (SeMet), which exhibit higher bioavailability compared to inorganic species [47]. Appropriate Se supplementation in soil has been shown to promote plant growth and increase crop yield when Se levels are relatively low (≤5 mg kg^−1^). However, excessive Se application (e.g., ≥10 mg kg^−1^) may lead to the accumulation of inorganic species such as Se(IV) and Se(VI) in grains, potentially posing risks to human health. Therefore, a Se application range of 0.5–5 mg kg^−1^ is generally considered safe and effective for the production of Se-enriched rice [45].

Hydride generation atomic fluorescence spectrometry (HG-AFS) has traditionally been used for total Se determination. However, due to the chemical selectivity involved in the hydride generation process, it can also provide limited speciation information. Under typical acidic conditions (e.g., 1–6 M HCl), only Se(IV) readily reacts with reducing agents to form volatile SeH_4_, whereas Se(VI) does not participate in the reaction unless it is first reduced to Se(IV), commonly by heating with concentrated hydrochloric acid [48]. This difference in reactivity makes it possible to distinguish Se(IV) from total inorganic Se following a pre-reduction step. Despite these advantages, HG-AFS is still primarily applied to total Se analysis and does not allow direct identification of individual Se species without prior separation. Moreover, reliable quantification generally requires complete sample digestion, and matrix effects may still influence the analytical results, particularly in complex samples.

### 3.2. Atomic Absorption Spectroscopy

AAS is a widely used technique for elemental analysis, employing different atomization modes, including flame AAS (FAAS), electrothermal atomization AAS (ETAAS, also known as graphite furnace AAS), and hydride generation AAS (HG-AAS) [49]. Among these, HG-AAS is particularly suitable for hydride-forming elements such as Se, As, Sb, and Te due to its high sensitivity and operational simplicity. Similar to AFS, HG-AAS can provide valuable information on Se speciation by controlling the reduction environment, allowing for the selective determination of Se(IV) versus total Se [50]. AAS offers several advantages, including good selectivity, relatively low cost, and straightforward instrumentation. However, its sensitivity is generally lower than that of AFS and ICP-MS, particularly for trace-level Se determination.

To overcome this limitation, various pre-concentration techniques—such as liquid–liquid microextraction, supramolecular solvent extraction, and ionic liquid-based methods—have been developed and successfully applied to food samples [29,31]. These approaches significantly improve detection limits and analytical performance. Nevertheless, accurate determination of ultra-trace Se concentrations remains challenging using AAS alone, and additional enrichment or separation steps are often required. Furthermore, matrix interferences and analytical uncertainties may affect the reliability of results, particularly in complex biological samples [51].

### 3.3. Inductively Coupled Plasma–Mass Spectrometry

ICP-MS is one of the most powerful and widely used techniques for the determination of trace elements in biological and food samples. It offers exceptional sensitivity, multi-element capability, and a wide dynamic range, making it a preferred method for total Se analysis [52]. However, the accuracy of Se quantification can be severely compromised by spectral interferences, most notably from polyatomic argon-based ions (e.g., ^40^Ar^38^Ar^+^, which interferes with ^78^Se^+^). The initial and critical step in method development is the judicious selection of the Se isotope (^77^Se,^78^Se, or ^80^Se) to minimize these inherent challenges [53]. To reduce spectral interferences, modern ICP-MS instruments are often equipped with collision/reaction cell technology. In collision mode, helium gas is commonly used to remove interfering species through kinetic energy discrimination. In contrast, reaction mode employs gases such as hydrogen or methane to chemically react with interfering ions, thereby reducing their impact and allowing more accurate detection of Se ions. The use of CRC significantly improves signal-to-noise ratios and can lower detection limits to the ng L^−1^ (parts-per-trillion) level.

Sample preparation for ICP-MS is relatively straightforward and typically involves dilution or chemical digestion using acids or alkaline solutions, often assisted by microwave or high-pressure systems to ensure complete dissolution of solid matrices [54,55,56,57]. ICP-MS has been extensively applied to a wide range of Se-containing samples, including cereals, nuts, meats, and plant-derived foods, demonstrating high accuracy and reliability [58,59,60,61,62]. Method optimization strategies, such as carbon enhancement through methane addition, have been shown to further improve sensitivity and reduce detection limits to the ng kg^−1^ level [58]. In addition, ICP-MS enables the investigation of Se distribution in biological tissues and the effects of dietary supplementation, with studies indicating that organic forms such as SeMet are more effective in increasing total Se accumulation in muscle tissues [62]. Despite these advantages, ICP-MS requires expensive instrumentation and skilled operation. It is also subject to potential spectral interferences, which necessitate careful method optimization and calibration to ensure accurate quantification.

## 4. Se Speciation Analysis

Gaining insights into the metabolic processes of Se in agricultural products and the potential health benefits of these Se species requires detailed information on individual Se species. Thus, Se speciation analysis is necessary. Several analytical approaches are commonly used for Se speciation, each with distinct strengths and limitations. A key consideration in Se speciation is the efficiency of extraction techniques and their ability to maintain the stability of Se species throughout the process.

From the available research, Se species exhibit varying metabolic pathways, bioavailability, and potential health risks depending on their chemical forms. For instance, Se in the form of organic compounds like SeMet and Sec is highly bioavailable and essential for the synthesis of selenoproteins, which play crucial roles in enzymatic functions and antioxidant defense. Organic Se species, especially SeMet, are typically incorporated into proteins and contribute to better bioavailability and lower toxicity compared to inorganic forms [36]. In contrast, inorganic forms like Se(IV) and Se(VI) require conversion within the body to be utilized and are generally associated with higher toxicity [34]. Additionally, different Se species are associated with distinct health impacts. While organic Se forms, such as those found in Se-enriched foods, are beneficial and can improve antioxidant activity and prevent diseases such as cancer, inorganic species tend to accumulate in tissues and may lead to toxicity at higher levels. Thus, understanding Se speciation is vital for evaluating its health benefits and risks in food consumption. More specifically, in the analysis of Se in food, various studies have employed advanced techniques such as HPLC-ICP-MS to accurately quantify these species, confirming the importance of Se’s chemical form in determining its bioavailability and impact on health.

HPLC-ICP-MS is widely considered one of the most reliable techniques, offering high sensitivity and precise separation of inorganic forms, such as Se(IV) and Se(VI), from organic species like selenomethionine [63]. However, while HPLC-ICP-MS is powerful, the extraction efficiency can be affected by factors such as the sample matrix and the specific Se species present, which makes careful sample preparation essential for accurate results. ESI-MS complements this by enabling structural identification of novel selenocompounds, although it is mainly qualitative and requires relatively pure extracts. This trade-off between sensitivity and purity highlights the need for effective extraction methods that minimize species interconversion [64]. Other hyphenated techniques, such as HPLC-AFS or GC-MS, can provide good sensitivity and selectivity, but they are less commonly applied due to the need for specialized instrumentation and method development.

Several approaches have been proposed for Se species extraction, including enzymatic, acidic, and basic hydrolysis. The choice of sample preparation depends on the sample matrix, the expected chemical form of Se, and the analytical technique used. As the first step, sample treatment must ensure quantitative extraction while preventing species interconversion. In biological samples, Se is mainly present as selenoamino acids, either incorporated into proteins or in free form [61]. Enzymatic digestion is the most commonly used method, as it converts protein-bound Se into soluble species. Its efficiency depends on factors such as enzyme type, pH, temperature, and extraction time, which must be carefully optimized to avoid species loss or transformation. Non-specific proteolytic enzymes (e.g., proteases) are widely used to break proteins into peptides and amino acids. After extraction, Se species must be separated, identified, and quantified [65].

The data presented in Table 2 highlight the complex nature of Se speciation in agricultural products, with different Se species exhibiting distinct nutritional and toxicological properties. The analysis of Se species in various Se-enriched foods, including rice, garlic, broccoli, and animal products, demonstrates the need for diverse detection techniques to provide a comprehensive understanding of Se’s bioavailability and safety. Enzymatic hydrolysis with proteases like trypsin, alcalase, and protease K is commonly used to extract Se from plant sources such as Cardamine violifolia, broccoli, and bamboo shoots, achieving extraction efficiencies up to 75% [66]. Similar protease treatments are applied to Se-enriched garlic, while microwave digestion is frequently used for both plant and animal samples before analysis with ICP-MS and other techniques. The limits of detection (LOD) for Se species, including SeCys_2_, methylselenocysteine (MeSeCys), Se(IV), SeMet, and Se(VI), range from 0.08 to 0.15 μg L^−1^ using RP-HPLC-ICP-MS and HG-AFS methods [46]. These methods typically use C8 or C18 columns with an isocratic or gradient elution at a flow rate of 1.0 mL min^−1^, with ionic liquids often employed as mobile phase modifiers to enhance peak resolution and separation efficiency. The Se species analyzed vary, with SeCys_2_ and SeMet being prevalent in plant-based foods, while Se-enriched soybean and *C. violifolia* exhibit higher concentrations of organic Se. These findings highlight the need for appropriate analytical strategies to ensure accurate Se speciation and reliable evaluation of Se biofortification.

### 4.1. High Performance Liquid Chromatography-Inductively Coupled Plasma-Mass Spectrometry

HPLC is the most widely used separation technique for Se speciation analysis due to its ability to separate thermally labile and nonvolatile compounds. It offers a wide selection of stationary phases and mobile phase systems, enabling flexible adaptation to different Se species. However, despite its excellent separation capability, the accuracy of Se speciation analysis still strongly depends on the efficiency and stability of the extraction step.

**Figure 3:**
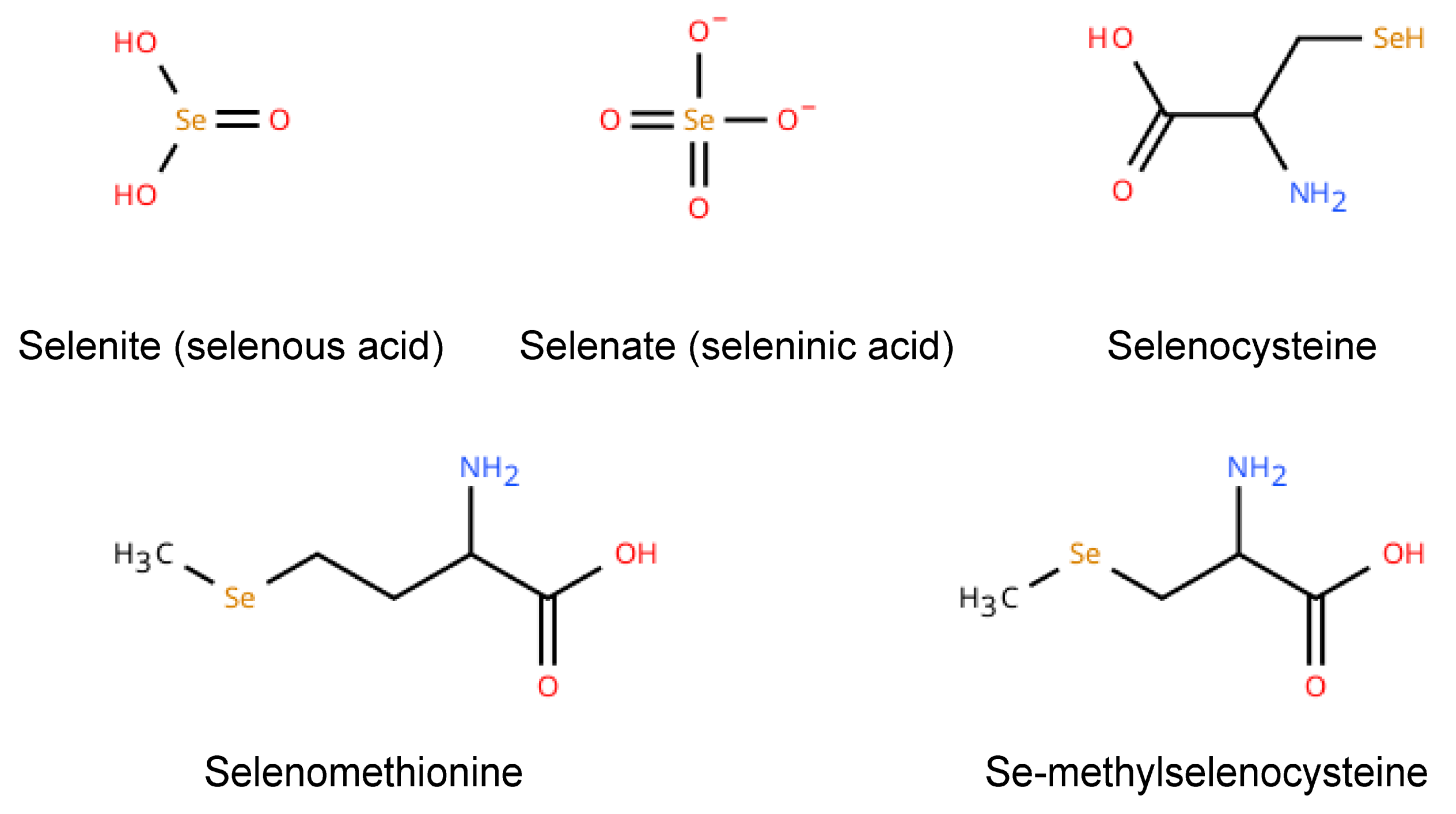
Structures of common Se species found in higher plants.

Due to the diverse chemical forms of Se present in food matrices, various liquid chromatography separation mechanisms have been developed and applied (Figure 3). The most commonly used modes include reversed-phase (RP), ion-exchange (anion or cation exchange), buffer concentration, and hydrophilic interaction liquid chromatography (HILIC) [73]. RP-HPLC primarily separates analytes based on hydrophobic interactions and is often combined with ion-pairing reagents to improve the retention of polar Se species [74]. Ion-exchange chromatography, especially anion-exchange mode, is widely used for separating inorganic Se species such as Se(IV) and Se(VI) based on their charge differences. Ion-pair chromatography integrates both hydrophobic and ionic interactions, allowing simultaneous separation of organic and inorganic Se compounds [75]. In addition, HILIC has attracted increasing attention for Se speciation because it provides improved retention and separation of highly polar Se metabolites and other hydrophilic Se compounds that are insufficiently retained on conventional RP columns [76]. The choice of mobile phase composition, including buffer type, pH, and ion-pairing agents, plays a critical role in achieving efficient separation and maintaining species stability [77].

Inductively coupled plasma mass spectrometry (ICP-MS) has been extensively applied to Se speciation analysis due to its high sensitivity and multi-element detection capability. However, all Se isotopes (^74^Se, ^76^Se, ^77^Se, ^78^Se, ^80^Se, and ^82^Se) are subject to isobaric or polyatomic interferences. For example, although ^80^Se has the highest natural abundance (49.6%), it suffers from significant interference from ^40^Ar_2_^+^. Therefore, isotopes such as ^78^Se, ^82^Se, ^77^Se, and ^76^Se, which have relatively lower abundance but reduced interference, are commonly monitored in practice [78,79,80]. HPLC coupled with ICP-MS has emerged as a powerful tool for Se speciation, enabling detailed investigation of Se metabolism in biological systems.

Zhang et al. [60] applied HPLC-ICP-MS to investigate Se speciation in pig muscle following different dietary Se treatments. The results showed that the deposition efficiency of Se sources followed the order: selenomethionine > methylselenocysteine > selenite. A dose–response relationship was also observed between dietary selenomethionine supplementation and muscle Se content. Four Se species were identified, with selenomethionine (>70%) and selenocystine (>11%) being the dominant forms, followed by methylselenocysteine and selenite. These findings demonstrate that both the form and level of dietary Se significantly influence Se speciation in animal tissues.

Bakırdere et al. [81] quantified Se species in chicken breast using HPLC–ICP-MS under optimized enzymatic hydrolysis conditions. The limits of detection (LOD) for Se(IV), Se(VI), SeMet, and SeCys_2_ were 0.75, 0.80, 0.55, and 0.46 ng mL^−1^, respectively. Protease XIV in Tris–HCl buffer (pH 7.2) was used for extraction. The total Se concentrations in the control, inorganic Se-fed, and organic Se-fed groups were 675 ± 85 ng g^−1^, 1084 ± 198 ng g^−1^, and 887 ± 139 ng g^−1^ (dry weight), respectively. Notably, SeMet was significantly higher in the organic Se-fed group, while Se(IV) was detected only in the inorganic Se-fed group.

Luo et al. [82] reported that SeCys_2_, MeSeCys, and SeMet were the dominant Se species in peanut proteins, and foliar Se fertilization enhanced the conversion of inorganic Se to organic forms. Similarly, studies by Warburton et al. [59], Duncan et al. [83], and Hu et al. [84] identified five major Se species in wheat, including Se(IV), Se(VI), SeCys_2_, MeSeCys, and SeMet. These studies highlight the versatility of HPLC–ICP-MS in analyzing Se species across diverse food matrices.

In recent years, Se nanoparticles (SeNPs) have attracted considerable attention due to their higher bioactivity and lower toxicity compared to inorganic and organic Se forms [85,86]. SeNPs are considered promising candidates for novel dietary Se supplementation and functional food development. However, their determination remains analytically challenging because of their particulate nature, instability, and potential transformation during sample preparation. Advanced techniques, such as coupling size-based separation methods (e.g., field-flow fractionation or size-exclusion chromatography) with ICP-MS, are being explored, but standardized analytical protocols for SeNPs in food systems are still lacking [87,88]. Notably, SeNPs have been detected in natural food samples, particularly in plant-based foods, suggesting that they may represent an important yet underexplored form of dietary Se [89]. In this context, single-particle ICP-MS (spICP-MS) has emerged as a powerful tool for the detection and quantification of SeNPs, enabling the determination of particle size distribution and number concentration at environmentally relevant levels [90].

Although SeCys is an important bioactive Se species present in plants and proteins, its direct determination in food samples is difficult. SeCys is highly reactive and readily oxidized during sample preparation and analysis. As a result, it is often not detected directly as a free species. To overcome this limitation, carbamidomethylation with iodoacetamide has been used to derivatize SeCys into a more stable form prior to chromatographic analysis. This stabilization does not preserve SeCys in its native form, but converts it into a defined derivative that can be more reliably detected, thereby improving confidence in inferring the presence of SeCys in natural samples.

Overall, among the various analytical techniques available, HPLC-ICP-MS combined with appropriate sample pretreatment remains the most representative and widely applied method for Se speciation analysis in food and biological samples.

### 4.2. Electrospray Ionization Mass Spectrometry

In recent years, it has become common to integrate electrospray mass spectrometry (ESI-MS, ESI-MS-MS) or matrix-assisted laser desorption ionization time-of-flight mass spectrometry (MALDI-TOF-MS) measurements into speciation studies. Both techniques offer a key advantage because they allow identification of species based on the isotope patterns [91].

While ESI-MS provides structural identification, it is mainly qualitative, which underscores the need for complementary quantitative techniques like HPLC-ICP-MS. Kotrebai et al. [92] reported the combined use of HPLC-ESI-MS for the quantitative and qualitative analysis of Se species in Se-enriched plants, such as the As-hyperaccumulating plant Tragalus praelongus or Brassica juncea, as well as in the different Allium varieties (garlic, *Allium sativum*; onion, *Allium cepa*; and ramp, *Allium tricoccum*).

Dernovics et al. [93] proposed a method for the identification of Se peptides in Brazil nuts. The protein extract was first fractionated by size exclusion chromatography. After tryptic digestion and pre-concentration of the selenized fractions, the peptides were identified by nano-HPLC-ESI-QTOF/MS/MS. Around 15 Se peptides were identified.

Warburton and Infante [58] reported, for the first time, the presence of selenomethionine in an enzymatic wheat flour extract based on accurate molecular mass data obtained by ion-pairing reversed-phase HPLC-ESI-Q-TOF-MS/MS measurements of the [M + H]^+ 80^Se ions and their corresponding product ions, without any pretreatment of the aqueous extract. Analysis of an enzymatic extract of bread made with Se-enriched wheat flour revealed selenate concentrations comparable to those observed for Se-enriched wheat flour (approximately 1%). Although SeMet was again found to be the predominant Se species, only 42% of the total Se concentration in bread was present as SeMet.

## 5. Systematic Evaluation and Analytical Challenges of Selenium Detection

The rapid advancement of Se biofortification necessitates robust analytical frameworks to accurately quantify both total concentration and chemical speciation (Table 3). Among current methodologies, AFS hyphenated hydride generation (HG) has emerged as a premier technique for total Se determination due to its high sensitivity and cost-effectiveness, making it suitable for routine screening [94,95]. While AAS provides broad accessibility and low operational thresholds—particularly with graphite furnace AAS achieving ppb-level detection—its sensitivity remains inferior to AFS and ICP-MS. Furthermore, both AFS and AAS are inherently limited to total Se quantification, as the atomization process precludes direct speciation analysis [96].

ICP-MS represents a superior alternative, characterized by multi-element detection capabilities and exceptional trace-level sensitivity, making it particularly suitable for complex matrices or low-concentration samples. The coupling of HPLC-ICP-MS provides a definitive platform for speciation, enabling the precise separation of inorganic forms, such as Se(IV) and Se(VI), from organic species like selenomethionine [97]. For molecular characterization and the identification of novel selenocompounds, ESI-MS serves as a critical qualitative tool. However, significant challenges remain in the analysis of agricultural matrices. These include variable extraction efficiencies, matrix effects that can compromise accuracy, and the potential for Se species interconversion during sample preparation or food processing. Moreover, the lack of certified reference materials limits validation, and proteomic approaches for selenoprotein identification are constrained by low intrinsic Se concentrations.

Overall, AFS and ICP-MS are suitable for total Se quantification, whereas HPLC-ICP-MS and ESI-MS are preferred for quantitative and structural speciation analysis, respectively [74,98].

## 6. Conclusions

In conclusion, the field of Se determination in agricultural products is evolving from routine total Se analysis toward integrated speciation-focused assessment. Recent progress has not only improved the characterization of conventional inorganic and organic Se species, but has also extended analytical attention to highly polar Se compounds, unstable intermediates, and Se nanoparticles. These advances underline the need for analytical strategies that better preserve species integrity and more accurately reflect the true Se composition of agricultural matrices. Future studies should therefore prioritize species-preserving extraction, standardized analytical workflows, appropriate reference materials, and a deeper understanding of less-characterized Se forms in real biofortified crops and plant-derived foods. Such efforts will strengthen the role of Se analysis in evaluating biofortification efficiency, food quality, and potential health benefits.

## Figures and Tables

**Table 1:** Examples of Total-Se in some agricultural products.

Product Category	Sample	Total Se	Technique	Reference
Dairy products and beverages	Milk	54.6 ± 3.5 μg L^−1^	GF-AAS	[28]
	Milk	61.7 ± 4.7 μg L^−1^	ICP-MS	[29]
	Beer	32.5 ± 1.8 μg L^−1^	ICP-MS	[29]
	Red wine	47.8 ± 3.5 μg L^−1^	ICP-MS	[29]
	Mixed fruit juice	75.2 ± 5.1 μg L^−1^	ICP-MS	[29]
Fruits and vegetables	Tomato	26.7 ± 1.82 μg kg^−1^	GF-AAS	[28]
	Apple	32.7 ± 4.27 μg kg^−1^	GF-AAS	[28]
	Apple	29.4 ± 1.7 μg kg^−1^	ICP-MS	[29]
	Orange	55.2 ± 4.2 μg kg^−1^	ICP-MS	[29]
	Grapefruit	39.6 ± 2.3 μg kg^−1^	ICP-MS	[29]
	Date	62.8 ± 4.6 μg kg^−1^	ICP-MS	[29]
	Cabbage ^a^	11,000 ± 2000 μg kg^−1^	HPLC-ICP-MS	[30]
	Cabbage ^a^	65,000 ± 4000 μg kg^−1^	HPLC-ICP-MS	[30]
	Cabbage ^a^	952,000 ± 16,000 μg kg^−1^	HPLC-ICP-MS	[30]
	Se-enriched garlic	250,000 ± 4000 μg kg^−1^	ICP-MS	[34]
Cereals and legumes	Rice flour	288.1 μg kg^−1^	HG-AAS	[31]
	Buckwheat flour	69.7 μg kg^−1^	HG-AAS	[31]
	Soybean	35.8 μg kg^−1^	HG-AAS	[31]
	Rice	38 ± 10 μg kg^−1^	HG-AFS	[35]
Eggs and meat	Turkey eggs ^b^	479 ± 206 μg kg^−1^	AFS	[32]
	Goose eggs ^b^	450 ± 117 μg kg^−1^	AFS	[32]
	Chicken eggs ^b^	374 ± 81 μg kg^−1^	AFS	[32]
	Egg	120.3 ± 8.6 μg kg^−1^	ICP-MS	[29]
	Beef ^c^	372.7 μg kg^−1^	Fluorimetric	[36]
	Lamb	350 ± 30 μg kg^−1^	ICP-MS	[37]
Fish and seafood	Canned fish	111 ± 8.43 μg kg^−1^	HG-AAS	[31]
	Canned shrimp	175.3 μg kg^−1^	HG-AAS	[31]
	Canned tuna	95.6 μg kg^−1^	HG-AAS	[31]
Other products	Laver	119 ± 12 μg kg^−1^	HG-AFS	[35]
	Spirulina	228 ± 60 μg kg^−1^	HG-AFS	[35]
	1-day-old mushroom	31,000 ± 4900 μg kg^−1^	ICP-MS/MS	[38]
	Black pepper	11,100 ± 500 μg kg^−1^	VA-IL-DLLME ^d^	[33]
	Tea	6.8 μg kg^−1^	HG-AFS	[39]
	Honey	90.4 ± 6.5 μg kg^−1^	ICP-MS	[29]
	Animal feed	10 μg kg^−1^	HPLC-UV-CVG-AFS	[40]

Note: ^a^ Cabbage seeds were grown in pots supplemented with three levels of Se sodium salts (Se(IV): Se(VI) = 1:9), corresponding to 6, 21, and 169 mg Se kg^−1^, respectively. ^b^ Yolk and albumen. ^c^ 2.7 mg of organic Se kg^−1^ of dry matter in bovine diet. ^d^ Vacuum-assisted ionic liquid-based microextraction and spectrophotometric analysis.

**Table 2:** Examples of Se speciation in some agricultural products.

Sample	Sample Preparation	Concentration of Se Species	Separation/ Detection	Reference
Se-enriched rice	Enzymatic extraction	Organic Se: 94.6–116.2 ng g^−1^; Inorganic Se: 18.2–29.6 ng g^−1^	HG-AFS	[43]
*C. violifolia*	Compound enzymatic hydrolysis with proteases in Tris–HCl buffer	SeCys_2_: 2154 μg g^−1^; MeSeCys: 18.5 μg g^−1^; Se(IV): 226 μg g^−1^; SeMet: 4.46 μg g^−1^; Se(VI): 52.3 μg g^−1^	RP-ICP-MS	[66]
Broccoli	Compound enzymatic hydrolysis with proteases in Tris–HCl buffer	SeCys_2_: 693 μg g^−1^; MeSeCys: 5.20 μg g^−1^; Se(IV): 98.8 μg g^−1^; SeMet: 4.59 μg g^−1^; Se(VI): 59.5 μg g^−1^	RP-ICP-MS	[66]
Cabbage	Compound enzymatic hydrolysis with proteases in Tris–HCl buffer	SeCys_2_: 2.70 μg g^−1^; MeSeCys: 20.8 μg g^−1^; Se(IV): 0.51 μg g^−1^; SeMet: 21.9 μg g^−1^; Se(VI): 94.7 μg g^−1^	RP-ICP-MS	[66]
Soybean	Compound enzymatic hydrolysis with proteases in Tris–HCl buffer	SeCys_2_: ND; MeSeCys: 1.93 μg g^−1^; Se(IV): 3.60 μg g^−1^; SeMet: 98.4 μg g^−1^; Se(VI): 11.3 μg g^−1^	RP-ICP-MS	[66]
Bamboo shoot	Compound enzymatic hydrolysis with proteases in Tris–HCl buffer	SeCys_2_: 49.0 μg g^−1^; MeSeCys: 22.2 μg g^−1^; Se(IV): 11.5 μg g^−1^; SeMet: 55.0 μg g^−1^; Se(VI): ND	RP-ICP-MS	[66]
Cabbage	Simulated gastric/gastrointestinal PBET extraction	At total Se levels of 11, 98, and 952 mg kg^−1^, Se(VI) was the predominant species in both gastric and gastrointestinal PBET extracts (2.8 ± 0.8–347 ± 12 mg kg^−1^). Lower concentrations of Se(IV) (0.48 ± 0.06–27 ± 1 mg kg^−1^), SeMet (0.7 ± 0.2–18.4 ± 2.4 mg kg^−1^), and SeCys_2_ (0.55 ± 0.06–3.3 ± 0.6 mg kg^−1^) were also detected.	Anion-exchange LC-ICP-MS	[30]
Garlic	Protease-assisted extraction in Tris buffer	Se(IV): ND; Se(VI): 97.9–158.9 μg L^−1^; SeMet: 476.8–819.9 μg L^−1^; SeMeSeCys: 954–2899 μg L^−1^	RP-HPLC-HG-AFS	[46]
Yeast	Protease-assisted extraction in Tris buffer	Se(IV): 9.6–11.5 μg L^−1^; Se(VI): ND–10.9 μg L^−1^; SeMet: ND; SeMeSeCys: ND	RP-HPLC-HG-AFS	[46]
Wine	Direct analysis after dilution and pH adjustment	Se(IV): ND–8.0 μg L^−1^; Se(VI): ND–6.9 μg L^−1^; SeMet: ND; SeMeSeCys: ND	RP-HPLC-HG-AFS	[46]
Beer	Direct analysis after dilution and pH adjustment	Se(IV): 8.4–11.6 μg L^−1^; Se(VI): ND; SeMet: ND; SeMeSeCys: ND	RP-HPLC-HG-AFS	[46]
Se-enriched strawberries	Ultrasonic-assisted enzymatic hydrolysis	Se(IV): 2.8 mg kg^−1^; Se(VI): 3.3 mg kg^−1^; SeMet: 22.6 mg kg^−1^; SeCys_2_: 2.9 mg kg^−1^; SeMeSeCys: 10.4 mg kg^−1^;	HPLC-TR-HG-AFS	[67]
Wheat Flour	Microwave-assisted enzymatic extraction with Protease XIV	Se(VI): 28.4 ± 1.2 ng g^−1^; Se(IV): 3.1 ± 0.8 ng g^−1^; SeCys_2_: 68.4 ± 3.1 ng g^−1^; SeMeSeCys: ND; SeMet: 935 ± 9 ng g^−1^	HPLC-DRC-ICP-MS	[68]
Rice	Ultrasonic alkaline extraction with KOH	Se(IV): 712.3 μg kg^−1^; Se(VI): 81.7 μg kg^−1^	HPLC-ICP-MS	[62]
Tea	Ultrasonic alkaline extraction with KOH	Se(IV): 3.9–17.9 μg kg^−1^; Se(VI): ND	HPLC-ICP-MS	[62]
Garlic	Ultrasonic alkaline extraction with KOH	Se(IV): 2.0–4.9 μg kg^−1^; Se(VI): ND	HPLC-ICP-MS	[62]
Mung bean	Protease-assisted extraction	124.8–615.5 mg kg^−1^	HPLC-MS	[69]
Pork	Artificial gastric juice extraction	SeMet: 404.4–6050 μg kg^−1^; SeCys_2_: 63.5–113 μg kg^−1^; MeSeCys: 10.7–40.0 μg kg^−1^; SeUr: ND–46.7 μg kg^−1^	HPLC-ICP-MS	[42]
Milk	Protein/enzymatic extraction	Total Se: 39–583 μg kg^−1^; SeMet: 13.0–530.4 μg kg^−1^; SeCys_2_: 15.6–22.2 μg kg^−1^;	HPLC-ICP-MS	[70]
Pork	Artificial gastric juice extraction	SeMet: 123.7 μg kg^−1^; SeCys_2_: 100.9 μg kg^−1^; Se(IV), Se(VI): ND	HPLC-ICP-MS	[71]
Chicken	Artificial gastric juice extraction	SeMet: 110.5 μg kg^−1^; SeCys_2_: 40.1 μg kg^−1^; Se(IV), Se(VI): ND	HPLC-ICP-MS	
Se-enriched yeast	Water extraction at elevated temperature	SeMet: 85.5–206.9 μg g^−1^; SeCys_2_: 50.8–74.4 μg g^−1^	RP-HPLC-ICP-MS	[72]
Se-enriched clover	Water extraction at elevated temperature	SeMet: 53.2 ± 0.9 μg g^−1^; SeCys_2_: 44.1 ± 1.4 μg g^−1^	RP-HPLC-ICP-MS	[72]

Note: Sample preparation is summarized according to the main extraction strategy and key extraction reagent/condition reported in each reference; detailed procedures can be found in the original studies. SeCys_2_, selenocystine; MeSeCys, methylselenocysteine; SeMet, selenomethionine; RP-ICP-MS, reversed phase—inductively coupled plasma mass spectrometry; SeMeSeCys, se-methylselenocysteine; HPLC-TR-HG-AFS, high-performance liquid chromatography-thermoreduction-hydride generation–atomic fluorescence spectrometry; HPLC-DRC-ICP-MS, high-performance liquid chromatography-dynamic reaction cell-inductively coupled plasma mass spectrometry; SeUr, Selenouracil.

**Table 3:** Critical Comparison of Analytical Techniques for Total Se Determination and Se Speciation Analysis.

Primary Application	Technique	Typical Limit of Detection	Advantages	Limitations & Challenges
Total Se analysis	AFS (mainly HG-AFS)	ng L^−1^/ng kg^−1^ level	Ultra-high sensitivity; good selectivity; low cost	Limited to total Se; determination; requires complete digestion and pre-reduction
	AAS (mainly HG-AAS/GF-AAS)	µg kg^−1^	Simple; widely available; low cost	Lower sensitivity than HG-AFS and ICP-MS; often requires pre-concentration
	ICP-MS	ng kg^−1^	Highest sensitivity; multi-element capability	Expensive instrumentation; skilled operation required; spectral interferences must be carefully controlled
Se Speciation analysis	HPLC-ICP-MS	μg L^−1^	High-resolution separation + sensitive detection; quantitative	Very high cost; sample preparation is critical to avoid species interconversion; ICP-MS-related spectral interferences remain a concern
	ESI-MS	Primarily qualitative	Structural identification; useful for unknown species	Limited quantitative capability; requires relatively pure extracts

## Data Availability

No new data were created or analyzed in this study. Data sharing is not applicable to this article.

## Abbreviations

The following abbreviations are used in this manuscript:

Se	Selenium
Sec	Selenocysteine
SeMet	Selenomethionine
SeCys_2_	Selenocystine
SeHoCys	Selenohomocysteine
SeMeCys	Se-methylselenocysteine
γ-Glu-MeSeCys	γ-glutamyl-Se-methylselenocysteine
AFS	Atomic fluorescence spectrometry
AAS	Atomic absorption spectroscopy
ICP-MS	Inductively coupled plasma mass spectrometry
HPLC-ICP-MS	High-performance liquid chromatography-inductively coupled plasma mass spectrometry
ESI-MS	Electrospray Ionization Mass Spectrometry
HG	Hydride generation
